# Father Involvement and Cognitive Development in Early and Middle Childhood: A Systematic Review

**DOI:** 10.3389/fpsyg.2019.02405

**Published:** 2019-10-25

**Authors:** Luca Rollè, Giulia Gullotta, Tommaso Trombetta, Lorenzo Curti, Eva Gerino, Piera Brustia, Angela M. Caldarera

**Affiliations:** Department of Psychology, University of Torino, Turin, Italy

**Keywords:** fathers, fathers involvement, cognitive skills, learning, parenting

## Abstract

This systematic review aims to examine the existing literature concerning the association between father involvement and the development children's cognitive skills during early and middle childhood. Specifically, it analyzes: (1) how the number of researches developed across years; (2) which are the main socio-demographic characteristics of the samples; (3) which are the main focuses examined; and (4) which operational definitions were used to assess father involvement and children cognitive skills. Following the guidelines of the Preferred Reporting Items for Systematic Reviews and Meta-Analyses (PRISMA) statement, the articles were searched through PubMed and EBSCO (PsycInfo, PsycArticles, Education Source, Social Sciences Abstract, Family Studies Abstracts, Gender Studies Database and CINAHL complete). The findings suggest that, although each research used a different operational definition of the father involvement construct, in recent years there was a wide and constant interest increase about this issue. Most of the empirical studies utilized quantitative methods, whereas relatively few used qualitative and only one mixed methods. As regards the analysis of socio-demographic characteristics of the samples there is a great evidence that most of them included biological and residential fathers: it may reflect that this type of sample is easier to recruit than non-residential and non-biological fathers. Regarding the socio-economic status and the ethnicity of families, the data highlighted how in recent years the literature on father involvement is starting to look at differences in ethnic and cultural backgrounds, in contrast to past researches. The findings revealed that the main focus is the impact of father involvement on children's cognitive skills and the most of the studies highlighted that it is positive and statistically significant. Regarding to the assessment of father involvement and children's cognitive skills, the literature is quite heterogeneous

## Introduction

Cognitive skills development is a broad concept that involves the maturing of a variety of abilities and is defined by the American Psychological Association (VandenBos, 2015) as “the skills involved in performing the tasks associated with perception, learning, memory, understanding, awareness, reasoning, judgment, intuition, and language” (p. 202). In this process, as Bandura highlighted in 1993, parents can play a crucial role, contributing to stimulating and supporting children's self-regulatory and cognitive development (Bandura, 1993). Indeed, parents who undertake verbal interactions and structure activities and games with their children allow them to live the rich linguistic communication and contexts of shared attention and meanings. These experiences encourage children's active exploration and engagement with their environments, improving children's academic skill and their success in school (Grossmann et al., 2002). Paternal involvement has been also situated within Bronfenbrenner's bioecological theory (1979) which highlighted that both proximal (e.g., paternal involvement) and distal (e.g., socioeconomic status, race/ethnicity, school context) factors must be considered to fully understand paternal influences on children's cognitive skills development. In line with these findings, various meta-analyses and reviews investigated the link between parental involvement during early and middle childhood and student achievement (e.g., Fan and Chen, 2001; Jeynes, 2007) and proximal and distal factors influence levels of fathers' involvement in literacy activities (e.g., Varghese and Wachen's, 2016). Even though various proximal and distal factors (e.g., fathers' education, income level, residencial status, relationship with the child's mother) are indirectly associated with children's language development and literacy activities (Varghese and Wachen, 2016), research confirmed a direct, positive and relatively strong association between paternal involvement and children's cognitive skills development (Fan and Chen, 2001; Jeynes, 2007). For instance, Varghese and Wachen (2016) found that fathers made unique and direct contributions to their children's literacy outcomes through their engagement in reading and writing activities and the use of complex language and responsive parenting behaviors.

However, notwithstanding the extensive literature on the topic, there is a great difficulty in finding an agreed definition of the construct of parental involvement, as it is conceptualized in a variety of ways (Harris and Goodall, 2007). For example, some authors distinguish between school- and home-based involvement: the first refers to the communications between parents and school personnel and to the parental engagement in activities children must perform at school (Grolnick and Slowiaczek, 1994; e.g., Hill, 2001), while the second (home-based) includes all the school activities that both mothers and fathers perform with their children at home (Hill, 2001). Other researchers investigated a further form of parental involvement not directly related to school (e.g., playing sports and other games, going to the cinema or a museum) that can influence school achievement (Nord et al., 1997).

Furthermore, while the first studies on the influence of parenting on children's outcomes focused mostly on mother–child dyadic interaction, nowadays, given the increasing number of working mothers and the changing social climate regarding male and female gender roles, the assumption that mothers were the gatekeepers and that father–child relationships had little impact on children's development is diminished. Consequently, various current studies are focused on the construct of coparenting as “the ways that parents work together in their roles as parents” (Feinberg, 2003, p. 1499) and to explore the determinants of fathers' involvement with their children's education (Lamb et al., 1985; Volling and Belsky, 1991). One of the most widespread and accredited models of the construct of father involvement was developed by Lamb et al. (1985). It includes: (a) engagement, the amount of time fathers spends in direct contact with their children (e.g., verbal stimulation, caregiving, and physical play); (b) accessibility, fathers' attendance and availability; and (c) responsibility, the ability to plan activities specifically adapted to the age and needs of the child (Lamb et al., 1987). Although this model allowed an increase in the understanding of the ways in which fathers are involved in their children's lives, it showed various gaps (Fagan et al., 2014). First, given the lack of systematic development regarding the different heuristic categories, there are only a few validated and reliable tools. Second, of the three types of involvement suggested by Lamb et al. (1985), researches have generally focused more on paternal engagement than on accessibility and responsibility, resulting in an overfocus on the amount of time fathers spend with their children (Pleck, 2012). As a consequence, Pleck (2010) widens the definition of “engagement,” identifying two distinctive elements: (a) positive engagement activities, the amount of time fathers and children are involved in interactive activities, such as reading books and teaching, that may promote learning and development; and (b) the dimensions of parenting, how fathers engage with their children, including the dimensions of Baumrind's (1967) parenting style model (*responsiveness and demandingness*). This contribution showed that the originally engagement construct may be not well-defined for studying the effects of fathers' parenting on children. However, many gaps related to the original tripartite model remained (Fagan et al., 2014). One of these gaps is that positive engagement was still largely focused on the amount of time fathers are involved with their children; another is related to the lack of a valid theory that guided the selection of the different dimensions of positive father engagement. In addition, the multidimensional construct has not been integrated into a comprehensive conceptual framework that could enable an understanding of what fathering is, why fathers parent in the manner they do, and how paternal actions directly and indirectly help determine children's development (Palkovitz, 2002; Pleck, 2007, 2010). To overcome this research gap, Cabrera and Volling (2019) proposed a “Developmental Ecological Systems Framework for understanding fathering and children's development.” This approach considers fathers as part of dynamic systems characterized by interconnected relationships between and among caregivers and children and explains how these relationships evolve and change through time and social and contextual factors (Cabrera et al., 2014, 2018). Previously, based on this ecological approach, Cabrera et al. (2014) developed a model to acknowledge the flow and actions affecting the quality (and quantity) of fathering on child functioning. According to this model, the father–child relationship results are influenced by the personality, personal characteristics, and behaviors of the father and other family members (e.g., mother, child, siblings) and the overall context of the family system by the family relationship (e.g., coparenting), the different family households, socioeconomic statuses (SES), and cultures, and the child's and family's development (Volling and Belsky, 1991; Cabrera et al., 2014; Cabrera and Volling, 2019). The strengths of this model are the inclusion of interpersonal and contextual variables in determining the level of father involvement (Volling and Belsky, 1991) and, also, the consideration of the transactional and reciprocal relationships between fathers and children (Sameroff, 2010). In line with complexity put in light by this model, the literature about father involvement is still very heterogeneous. As a consequence, in order to design innovative and rigorous research, it becomes necessary to increase knowledge about what has been previously investigated, particularly in relation to the following issues: how should father involvement, especially in early and middle childhood, be conceptualized and measured? What is the relationship between the different components of father involvement and children's cognitive skills? Which are the main focuses investigated? Are non-residential, step-, low-income, and minority fathers included in the research samples? How is it possible to increase father involvement in children's education?

Although some attempts of synthesis have already been produced, many are prior to 2010 (Saracho, 2007a,b; Downer et al., 2008), while the more recent are focused on specific themes, such as educational achievement (Lipscomb, 2011; Jeynes, 2015; Kim and Hill, 2015), and none were published after 2016. Consequently, the previously presented questions call for a systematic examination of the existing research concerning the association between father involvement and children's cognitive skills during early and middle childhood, to widen our knowledge about the definition and measurement of father involvement, the pathways of its influence on children's cognitive skills development, and the differences from/similarities to mother involvement.

## Aims

The aims of the present review are to systematically examine the literature about father involvement in the development of children's cognitive skills and, in particular, to analyze: (1) how the number of researches and the design/methods developed across years; (2) if non-residential, step-, low-income, and minority fathers are included in the research samples; (3) which are the main focuses of the examined literature; (4) which operational definitions of the construct and measures were used to assess father involvement and children's cognitive skills.

## Methods

### Data Source and Search Strategy

We followed the guidelines of the Preferred Reporting Items for Systematic Reviews and Meta-Analyses (PRISMA) statement (Moher et al., 2009). Articles were searched through PubMed and EBSCO (PsycInfo, PsycArticles, Education Source, Social Sciences Abstract, Family Studies Abstracts, Gender Studies Database, and CINAHL Complete).

**Figure 1:**
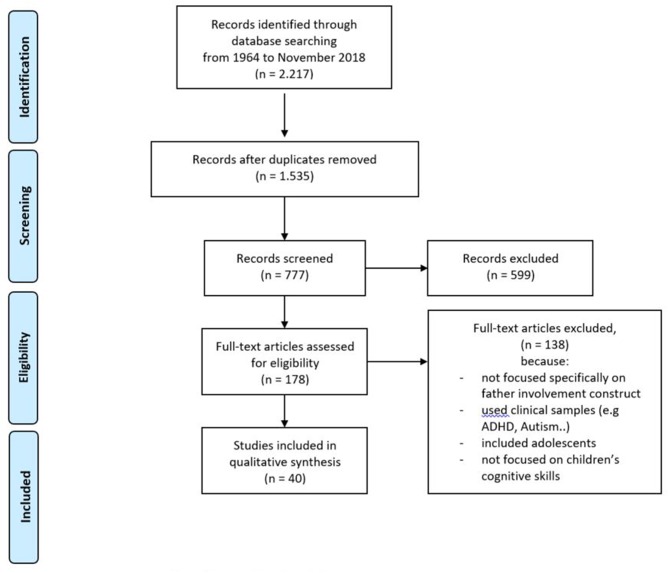
PRISMA flow diagram of study selction.

We examined titles and abstracts to find eligible studies published in English, from the first publication in 1964 to November 2018. The keywords used for the query were “Father^*^” and “learning” or “cognitive skills” and “children.” Of the 2,215 papers resulting from this first search, after duplicates removal, 777 met the criteria of being published in English in peer-reviewed journals. We selected, by titles and abstracts, 178 papers. As a second step, we selected the papers to be included in the data synthesis by reading the full-text. A total of 40 papers was finally retained (see Figure 1).

### Study Inclusion and Exclusion Criteria

The inclusion criteria for the papers were: (a) the presence of the father involvement construct and a specific reference to cognitive skills; (b) age of children not above thirteen; (c) written in English; and (d) published in academic journals. Both qualitative and quantitative articles were selected as well as reviews, with the aim of looking at the different approaches and methodologies used in this field.

The exclusion criteria were: (a) clinical samples to avoid bias in focusing more on father involvement as related to the outcomes associated with disease (e.g., ADHD, autism); (b) studies that were not specifically focused on the father involvement construct; (c) adolescent samples (2 studies with 10-year-old children were excluded because they were part of samples aged between 10 and 16 years); and (d) studies focused on other children's outcomes (e.g., physical, diet). Two independent reviewers selected the articles by title and abstract. Afterwards, they compared the results to determine a common list of selected papers; each choice that was different between the two reviewers was discussed in order to find an agreement.

### Strategy for Data Synthesis

The selected articles were independently entered by each reviewer into two classification tables: one dedicated to analyzing the reviews and meta-analyses, and the other to analyzing the qualitative, quantitative, and mixed-methods studies. The papers that, when reading the full text, did not match our selection criteria were further excluded. Each reviewer independently completed the tables, and discussed each discrepancy in the selection in order to find an agreement.

We subsequentely developed a dataset of the selected papers and conducted a thematic analysis (TA) with the purpose of defining the main focuses of the reviewed articles, using a semantic approach, according to the guidelines provided by Braun and Clarke (2006). The reviewers discussed the TA results as well.

To avoid a thematic overlap, each article was linked to a single topic, in particular, the main focus of each study.

## Results

Table 1 shows the frequency of the different types of papers. Quantitative methods were mostly used, whereas there were few qualitative and only one utilized mixed methods.

**Table 1:** Type of document.

**Study design**	**Frequency**	**Percentage %**
Quantitative	16	40
Qualitative	9	22.5
Meta-analysis	3	7.5
Review	4	10
Case report	5	12.5
Theoretical article	1	2.5
Mixed methods	2	5
	40	100

Table 2 (meta-analyses and reviews) and Table 3 (empirical studies, case reports, and theoretical articles) describe the researches' primary characteristics—authors and year, study design, objective, content focus, determinants, and key findings—in relation to general information and our review question. Other features of the reviewed literature will be discussed in detail in the following four paragraphs: growing interest with the role of father involvement in children's education, and the samples' sociodemographic characteristics, main focuses, and measurement methods.

**Table 2:** Reviewes and meta-analysies included in the study.

**Study and year**	**Study design**	**Objects**	**Variables**		**Focus**	**Key findings**
			**Determinant**	**Outcomes**		
Saracho (2007a)	Review	Hispanic father involvement in children's literacy development	Ethnicity minority	Literacy skills	FI and children's outcomes	Hispanic father support their children's literacy skills by (a) reading books (b) books discussion (c) recording book already read and (d) stimulating children to enhance their reading
Jeynes (2015)	Meta-anlysis	Relationship between father involvement and children's educational outcomes	Mix ethnicity Biological fathers Ages 3–20	Academic skills Psychological Welfare Positive behaviors Other healthy results	FI and children's outcomes	Relationship between father involvement and child educational outecomes is significant statistically both for white and minority children
Downer et al. (2008)	Meta-analysis	Father involvement and children's early learning	Mixed Ethnicity Mixed SES Mixed biological status Mixed residential status Ages 0–6	Academic and socio-emotional competence	FI and children's outcomes	The consistent of the association between father involvement and children's academic achievement is manteined across etnicity and SES
McWayne et al. (2013)	Meta-analysis	Father involvement and children's early learning	Mixed ethnicity Mixed SES Mixed biological status Mixed residential status Ages 3–8	Social and cognitive domains	FI and children's outcomes	Father involvement showed a consistent association with early childhood competencies, differing based on father's characteristic
Saracho (2007b)	Review	The role of father in supporting their children's literacy learning		Literacy skills	FI and children's outcomes	Fathers' contribution improve their children's literacy and academic skills
Kim and Hill (2015)	Meta-anlysis	The association between parental involvement and children's acedemic achievement	Mixed Ethnicity Mixed SES Mixed Residential status Ages 5–18		FI and children's outcomes	Parental involvement and student achievemente are positively, althought mothers' involvement is higher than fathers'
Lipscomb (2011)	Review	The effects of FI on their children's educational achievement and programs to increase it			FI and children's outcomes and intervention	There are several programs, particularly aimed to specific population or more general, that can increase father involvement in children education

**Table 3:** Studies included in the meta-analysis listed by author, year of study, type of document, objects, and other.

**References**	**Study design**	**Objective**	**Methods**		**Variables**		**Focus**	**Key findings**
			**Participants**	**Measures**	**Determinant**	**Outcomes**		
Potter et al. (2013)	Case report	Evaluete the benefits of Father Transition Project (FTP)	5 fathers and 2 grandfathers Children Ages 5–6	Interview Focus Group	Low SES	Enjoyment Achievement Learning	Intervention	Key benefits: a closer relationships with children and an improved involvement both in educational activities
Potter et al. (2012)	Case report	Assess thestrategies most successful in engaging fathers of FTP	5 fathers and 2 grandfathers Children Ages 5–6	Interview Focus Group	Low SES	Enjoyment Achievement Learning	Intervention	The most effective strategies were a personalized, strengths-based within a cooperation context and utilizing an empowerment approach.
Eng et al. (2014)	Quantitative	The role of social capital as a predictor of parental involvement in children's education	273 parents Children Ages 6–10	FI at school: P-TIQ (Parent Version) Walters (2001) FI at Home: Self-report	Minoriy Ethnicity		Determinant	Parents' social networks, academic ambition, trust, gender attitudes and fatalistic convinction can be considered as a predictor of parental involvement
Keown and Palmer (2014)	Qualitative	Compare father-son and mother-son involvement	94 families Children Age 4 (T1) Age 7 (T2)	Interview Questionnire Observation	Resident Fathers Mixed Ethnicity Mixed SES		Comparison	Mothers are more accessible to their son on the working days than fathers, while fathers spend more time with their children on weekend days
Flouri and Buchanan (2004)	Quantitative	Early father and mother involvement and child's later educational outcomes	3,303 parents and childrn Time 1 (age 7) Time 2 (age 20)	Self-report		Accademic Motivation General Ability	FI and children's outcomes	Father involvement indipendently and significantly predicted educational attainment by 20 years
Fagan and Lee (2012)	Quantitative	Effects of fathers and mothers cognitive stimulation and household income on single mother e two parents household on young childrend	8,400 children and parents 9 months (T1) 24 months (T2)	Mother Interview Fathers SelfReport Observation	Mixed SES Biological Fathers Mixed Residential Status	Cognitive skills (BSF)	FI and children's outcomes	Positive association between fathers' cognitive stimulation and children cognitive skills is stronger for children living in single mother household than for children living in 2 parents families
Coley et al. (2011)	Quantitative	Relationship between fathers early parenting and cognitive skills	261 biological Fathers Children Ages 2–4	Self-report	Mixed Ethnicity Low-SES Mixed Residential Status	Math reading (WJ-R)	FI and children's outcomes	Fathers' support and warm predicted higher accademic skills, over and beyond the chareteristics of the family
Baker (2018)	Quantitative	Father–school involvement and children's academic and social-emotional skills	3,570 children in kindergarten	Self-report	Mixed Ethncity Mid to high SES Mostly non residential fathers (74%)	Reading math and approch to leaning	FI and children's outcomes	Although mothers are more engaged in school involvement, father-school involvement is positively associated with children's academic skills
Chawla-Duggan (2006)	Qualitative	How father development workers supported fathers to increase paternal involvement in children's learning	4 fathers and their early years sons	InterviewFocus Group		Children's learning	Intervention	Father development workers support fathers within the group, raising confidence and responsibility, and with the child, improving children's learning
Black et al. (1999)	Mix methods	Low income fathers and competences, behaviors and home environment (HE) of preschool children	175 3-years African American Children	Self report “Who Does What” (Cowan and Cowan, 1988, 1990) Observation	Low-income Minority Ethnicity Mixed biological and residential status	Children's well-being: cognition, receptive language, behavior HE	FI & children's outcomes	There is a significant relationships between paternal role and each index of children's well-being.
McBride et al. (2013)	Quantitative	Examine the relationship between father involvement in school and children's achievement	596 children Ages 5–12 (T1)	Self-report	Mixed biological status Resident fathers Mixed ethnicity Mixed SES	Reading math (WJ)	FI and children's outcomes	It can be see a variation based on children gender, ethnicty and SES in the relationship between father involvement and children's achievement
Ball (2009)	Qualitative	To develop a theoretical framework about the experiences of Indigenous fathers in various needs and goals.	80 fathers Children Age under 7	Self-report Interview	Biological fathers (84%)		Determinant	Six ecological and psychological factors: personal well-being, learning, socio-economic inclusion, social, legislative and policy support and cultural continuity
Baker (2017)	Quantitative	The role of ethnicity and poverty status as a moderators of the association between father involvement and sons' cognitive and socio-emotional skills	4,240 young boys Ages 0–5	Self-report	Mixed Ethnicity Mid to high SES Mostly residential fathers (82%)	Math and reading	FI and children's outcomes	Paternal warmth and home learning stimulation (HLS) at T1 positively predicted cognitive and social emotional skills at T2, across raical groups.
Baker et al. (2018)	Quantitative	Relationship between family poverty, warmth and home learning stimulation (HLS) and children's preschool achivement	7,700 children Ages 0–5	Self-report	Mixed SES Mixed Ethnicity Biological and residential fathers	Reading and math	FI and children's outcomes	Although poverty negatively influences more fathers' parenting than mothers', fathers involvement turned out as stronger moderator between poverty and children's cognitive skills than mothers'
Tan and Goldberg (2009)	Qualitative	Parents involvement in children's education at school and at home	91 families and children Ages 6–10	Self-report (API) (Tan and Goldberg, 2009)	Biological (87%) Residential (92%) Non minority (74%) Mid to high SES	School Attitudes (SAS)	Comparion	Mothers' and fathers' school involvement show a different association with their sons' and daughters' sacademic achievement.
Giallo et al. (2013)	Quantitative	Fathers vs. mothers in the relationship between child, parents, family factors, parental involvement, and self-efficacy	851 mothers 131 fathers of children Ages 0–4	Self-report (PIS)	Non minority Mixed SES		Comparison	There are few differences between mothers 'and fathers' involvement. Parenal self efficacy plays a mediating role both for mothers and fathers
Bradley and Corwyn (2000)	Qualitative	Personal and contextual factors correlate with socioemotional investment in children.	65 fathers children Ages 0–2	PIC	Non minority Ethnicity Mid to high SES Biological and resident	Cognitive skills (MDI) (Bayley, 1993)	Determinant	Paternal involvement is multi-determined. There is no single factor that has a mastery role.
Foster et al. (2016)	Quantitative	Relationship between home learning environment (HLE) and children's academic skills	767 parents and children Ages 2–6	PQ (Morrison and Cooney, 2002)	Mid to high SES Ethnicity (80.4%) Biological fathers (99%)	Decoding non-minority (WJ-III) Letter Knowledge Math skills (TEMA-3)	FI and children's outcomes	Fathers involvement increase children's academic achievement only whether mothers have at most a high school diploma
Saracho (2008)	Case Report	Effects of Literacy Program, assisting fathers to support children's literacy skills	25 fathers and children Age 5	Interview Observation Documentary Analysis		Literacy skills	Intervention	In the program fathers learn literacy strategies to support and increase their children's literacy development
Kelly (2018)	Theoretical article	Conceptual model on the relationship between fathers engagement and children's prosocial skills				Cognition Emotional Regulation and social behaviors	FI and children's outcomes	Fathers engagement with their children is directly related to the childrend's cognitive skills, self-regulation and social behaviors, influencing civic readiness development
Anderson et al. (2015)	Qualitative	How the experience on Early Childhood Program (ECP) impact the father-role construction and support the engagement	7 fathers Ages 0–4	Focus Group	Low-income Biological resident (86%) Mixed ethnicity	Learning Enjoyment	Intervention	ECP supports fathers to develop parenting skills. These competences can improve father engagement and create a positive father–child relationships, changing the father-role construction.
Roopnarine et al. (2006)	Quantitative	The association between parenting styles and parent involvement and children's academic achievement and social behaviors	70 parents and children Ages 3–6	Interview Self-report	Minority ethnicity Mixed SES	Academic skills (K-SEALS) (Kaufman and Kaufman, 1993)	FI and children's outcomes	Father-school involvement is positively associated with children's academic competences but it is negatively associated with authoritarian parenting style
Hernandez and Coley (2007)	Quantitative	Psychometric properties of father and mother reports of father involvement	227 parents and children Ages 2–4	Self-report Mother-report	Low-income Minority ethnicity Mixed residential status Biological fathers	Cognitive skills (WJ-R)	Assessment	The reliability is similar between father and mother reports and among residential status and race.
Jeong et al. (2016)	Quantitative	Paternal stimulation and Early Child Development (ECD)in low- and midlle-income countries (LMICs)	87,286 children Ages 3–4	Mother-report	Residential Biological Mixed ethnicity and SES	Physical growth child development (ECDI)	FI & children's outcomes	When fathers are unengaged children have a lower ECD scores than children whose fathers highly engaged
Nordhal et al. (2016)	Qualitative	Predictors of fathers positive involvement and negative reinforcement	726 fathers Children Ages 0–1	Interview Observation NICHD (Cox and Crnic, 2003)	Mixed SES		Determinant	Positive involvement and negative reinforcement can be considered two different parenting dimensions
Sun et al. (2018)	Quantitative	Fathers engagement in early learning activities ss a protective factor in LMICs	7,583 children Ages 3–5	Mother-report	Ethnic majority Mixed SES	Early child development (EAP-ECDS)	FI and children's outcomes	Parenatal engagement moderates the relationship between SES and early learning
McBride et al. (2009)	Quantitative	Direct and indirect effects of early parenting on later parental school involvement and children academic achievement	390 children Ages 2–5 (T1)	Self-report	Resident Mixed biological status Non minority Mixed SES	Academic achievement (WJ)	FI and children's outcomes	Early parenting is significantly linked to later parental school involvement for both parents but it is not directly associated to academic achievement for both of them.
McBride et al. (2005)	Quantitative	Fathers school involvement as a mediator in the relationship between school, neighborhood family and children's academic skills	1,334 families Children Ages 5–12	Self-report	Resident Mixed biological status Non minority Mixed SES	Math Reading (WJ)	FI and children's outcoms	Father involvement result a mediator of the relationship between contextual factors and children's academic achievement.
Duursma (2014)	Qualitative	Paternal and maternal bookreading frequency and young children's language and cognitive development	430 families Children Ages 2–5	Interview	Low-income Mixed biological and residential status Mixed ethnicity	Cognitive skills (MDI) (Bayley, 1993) Language Development Literacy skills	FI and children's outcomes	Paternal bookreading significantly related to children's language and cognitive skills, although mothers read more than fathers.
Ortiz (2000)	Qualitative	Mexican American fathers bookreading frequency	25 father Children Ages 5–7	Questionnaire interviews Participant observation	Minority ethnicity Mixed SES	Literacy skills	FI and children's outcomes	Fathers involvement in early reading activities with their children, although the time varied by different area.
Cabrera et al. (2011)	Mix methods	Father engagement across race, monitoring the following variables: fathers' education, personal wellness, marital status and couple conflict	5,089 families Children Ages 0–1	Mothers Interview Fathers Self-report	Biological and resident fathers Mixed ethnicity and SES		Determinant	Fathers' education, marital status, couple conflict, depressive symptoms and type of involvemen do not differ by race, while physical pla and the levels of engagement change.
Baskwill (2008)	Case study	Program for increase fathers' perseptions of their role and their responsability in children's literacy development	15 fathers Children Ages 3–5		Mixed biological status Mixes SES Non minority Ethnicity	Litaracy skills	Intervention	During the program fathers can learn the importance of FI, devolop a repertoire of strategies as well as raise a confidence in their ability to engage in children education.

### Growing Interest in the Role of Father Involvement in Children's Education

This paragraph's main objective is to expand on the state of the literature about father involvement in children's cognitive skills development. One of the more widespread problems was that, in the literature on caregiving and children, fathers' parenting has been studied less than mothers' (Downer et al., 2008).

**Figure 2:**
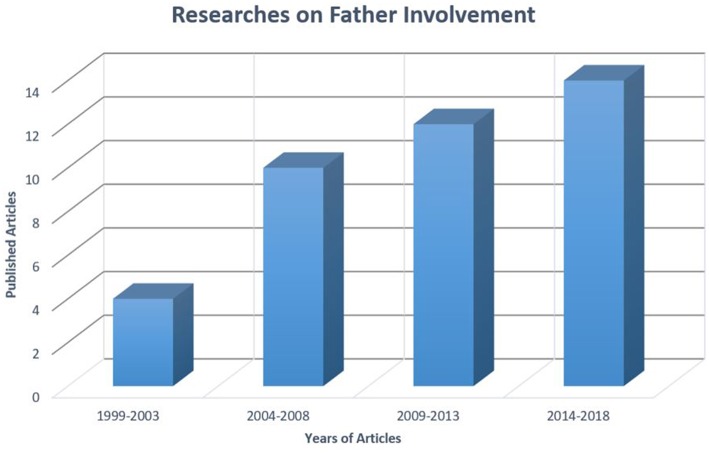
Published articles (included in this review) across years.

However, in the 70s and 80s the scholarly interest in fatherhood grew (Lamb, 2004) and, during the 90s, there was an increase in the researches on various fatherhood aspects, developing a large and heterogeneous body of studies that emphasized the unique role of fathers in children's development. For example, Marsiglio et al. (2000), in their review about fatherhood, examined the relationships between the dimensions of the father–child relationship (e.g., time spent with children, emotional support, everyday encouragement, and overseeing children's behaviors) and children outcomes. The increasing trend of the number of articles specifically focused on the construct of “father involvement” in children's education is shown in Figure 2. In the next paragraphs, we will focus on the main sociodemographic characteristics and measurement methods used in this growing literature.

### Samples' Demographic Characteristics

Regarding age, father involvement research was mostly conducted on groups of children aged 0–6 (27.5%), preschoolers (3–5) (22.5%), and infants/toddlers (0–3) (20%); mixed age (7.5%) and middle childhood samples (15%) were less frequent. However, reviews and meta-analyses focused on a wider age range, from early years to 20 years old.

Concerning fathers' characteristics, it is possible to see a predominance of biological and residential fathers: 35% of the reviewed studies collected samples of only biological fathers and more than 35% of only residential fathers. This trend may reflect that this sample type is easier to recruit. Indeed, only two studies used samples mostly comprised of non-residential fathers (Black et al., 1999; Baker, 2018). In the first quantitative study Baker (2018) investigated, in a sample of 74% non-resident fathers, father–school involvement was a predictor of improving academic achievement and social-emotional skills.

The results showed that, although mothers were always more engaged in their children's education, father–school involvement was positively associated with children's math and reading skills and with teacher-related approaches to learning during early childhood. In the second study with a sample composed of non-residential biological and non-biological African American fathers (who were involved at least monthly). Black et al. (1999) assessed the relationship between paternal roles (e.g., nurturance, emotional and economic support) and children's well-being.

The results showed that children whose fathers are satisfied with their parenting and economically supportive of their families have better language competences and cognitive skills, proving the unique fathers' contributions.

Nevertheless, regarding the families' SESs and ethnicities, we found a great heterogeneity across all the studies. Between the studies that distinguished minority, non-minority, and mixed ethnicity samples, 12.5% of the selected articles recruited only minority fathers, 30% exclusively focused on non-minority men, and 40% had a mixed ethnicity sample. More specifically, most of the studies that focused on minority or mixed ethnicity included African American (Black et al., 1999; Cabrera et al., 2011) and Hispanic fathers (Ortiz, 2000; Saracho, 2007a, 2008).

Despite the well-established relevance of household income for children's academic success, the research and knowledge about father involvement and children's cognitive skills in low-income families were lacking. Among the reviewed articles that studied the SESs of the families with children in early and middle childhood, 20% centered on low-income fathers while 42% included a mixed sample with family income ranging from low to high.

### Main Research Focuses

**Figure 3:**
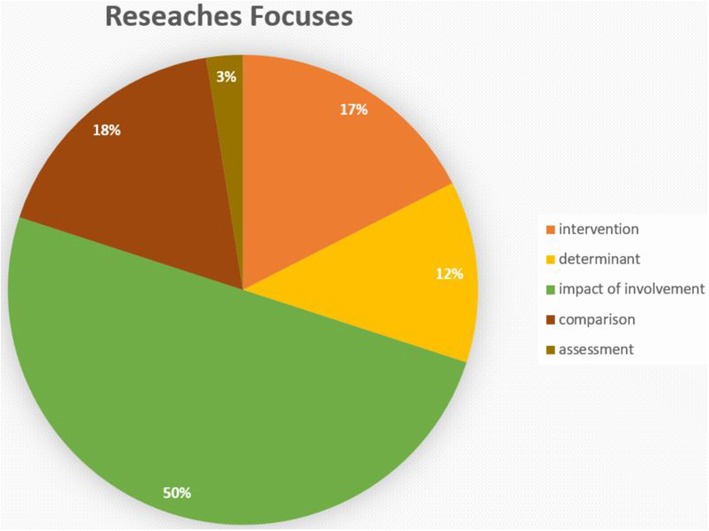
Main researches focuses.

As shown in Figure 3, half the empirical studies and all the reviews and meta-analyses focused on the relationship between father involvement and children's outcomes; a good number also focused on the effectiveness of interventions to increase fathers' engagement with their children's education and on the comparison between mother and father involvement, while a few articles examined the determinants, and only one study addressed the issue of assessment. In the following subparagraphs, we will synthetize the literature for each research focus, except the assessment, which we will discuss in the next paragraph.

### Extent of the Association Between Father Involvement and Children's Cognitive Skills

The findings of the reviewed articles (including reviews and meta-analyses), focusing in particular on the association between father involvement and children's cognitive skills, showed a positive and statistically significant association during early childhood (Flouri and Buchanan, 2004; McBride et al., 2005, 2009; Roopnarine et al., 2006; Saracho, 2007b; Downer et al., 2008; Coley et al., 2011; Fagan and Lee, 2012; McWayne et al., 2013; Duursma, 2014; Jeynes, 2015; Kim and Hill, 2015; Baker, 2017, 2018) beyond mothers' parenting (Roopnarine et al., 2006). More specifically, Fagan and Lee (2012) argued that this relationship was significantly strengthened for children living in single-mother households, whereas Coley et al. (2011) found that family characteristics were trifling.

However, it is important to underline that several studies highlighted that this pattern generally remains significant across ethnicity and SES (Downer et al., 2008; Jeynes, 2015; Baker, 2017).

For example, Cabrera et al. (2011) examined father engagement—verbal stimulation, caregiving, and physical play—across race, monitoring marital status, parental conflict, parental education, and depressive symptoms. The findings showed that while Caucasian fathers demonstrate a lower involvement in care and physical play than African American and Latino fathers, there were no differences in verbal stimulation across ethnicity. Furthermore, fathers' education (i.e., college level) was linked with more verbal activities, whereas couple conflict was associated with less engagement in care and physical activities. In conclusion, the authors argued that, although the level of involvement differed across ethnicity, the general model did not change.

Conversely, some scholars found that the relationship between father involvement and children's cognitive skills diverges across children's and parents' gender (Tan and Goldberg, 2009; Eng et al., 2014), race, and SES (McBride et al., 2013).

For example, Baker et al. (2018) found that, although poverty negatively influences more fathers' than mothers' parenting, fathers' engagement was a stronger moderator between poverty and children's cognitive skills than mothers' involvement.

### Comparison Between Maternal and Paternal Involvement

In addition to the studies that specifically focused on the relationship between father involvement and children's outcomes, another great part of research aimed at comparing maternal and paternal involvement in children's education. Most studies on this topic highlighted that, although the mean level of mothers' involvement was higher than fathers' (Duursma, 2014; Kim and Hill, 2015; Baker, 2018), fathers' involvement and children's academic skills had a positive association.

For example, Keown and Palmer (2014) collected a sample of 94 two-parent families, with the aim of comparing father and mother involvement with their young son. The findings revealed that, although the mothers were more available to their sons on workdays, and fathers were more involved in activities with their children on weekend days, both mother– and father–child conversations were rich resources for children during their school ages.

Another study, specifically focused on the comparison between maternal and paternal involvement, was conducted by Foster et al. (2016). The authors investigated the home learning environment (HLE) during early childhood and how mothers' and fathers' parenting practices predict children's academic outcomes. The findings showed that mothers provided HLE activities more frequently than fathers although, in families in which mothers had at most a high school diploma, fathers' contributions were a significant predicator of children's early academic skills. However, other studies indicate that also fathers' education levels were often associated with mother engagement and that parental education indirectly influences children's language development through multiple pathways (Pancsofar et al., 2010).

Furthermore, Duursma (2014) examined the association between book-reading frequency of low-income fathers and mothers and children's cognitive and literacy skills and found that, although mothers read to their children more frequently than fathers, approximately 55% of fathers reported that they read to their children weekly. Moreover, this study highlighted that fathers' book reading significantly predicted children's language competences, book knowledge, and cognitive skills.

In line with mentioned studies, Giallo et al. (2013) found quite a few differences between mothers' and fathers' involvement, despite both of them were influenced by parenatal self-efficacy (PSE), considering it one of the determinants of father involvement.

### Determinants of Father Involvement

Another group of articles specifically focused on the determinants of father involvement, examining both contextual, and personal factors.

Ball (2009), in a qualitative study about the involvement of 80 Canadian Indigenous fathers, introduced a conceptual model that identified six key ecological and psychological factors of fathers' involvement in circles of care for children. The six found factors are: (a) individual health, (b) learning fathering (direct father–child interaction, role models, and direct instructions), (c) socioeconomic inclusion, (d) social support for positive involvement, (e) legislative and policy support for involvement, and (f) cultural continuity.

In the same direction, Bradley and Corwyn (2000) investigated the influence of factors related to context (e.g., income), child characteristics (e.g., temperament), marital quality, and mother's and father's occupation on paternal socioemotional investment toward their children. The results highlighted that paternal investment is a multidetermined construct: it was not possible to identify one single factor that played a predominant role.

In recent years, Nordhal et al. (2016) suggested a conceptual model based on the “social interaction learning model” (SIL; Patterson, 1982), regarding the protective and risk factors of father involvement. Given that the interactions between children and parents can be considered a two-way process (Patterson, 1982; Patterson and Fisher, 2002). Nordhal et al. (2016) distinguished protective factors for fathers, including mental functioning and contextual and personal resources, and protective factors for children, including typical development, male gender, and easy behavior. While the negative factors related to fathers were mental dysfunction and personal and contextual strains, and the negative factors for children were development difficulties, female gender, and difficult behaviors. According to the findings, given that protective and negative factors were positively associated to fathers' positive involvement and to negative reinforcement, respectively, these last two dimensions could be considered different parenting components.

Another study that focused on the determinants of father involvement in Cambodian American families showed that parents' social networks, academic ambitions, trust, gender attitudes, and fatalistic convictions can be considered predictors of parental involvement (Eng et al., 2014). As a consequence, the authors highlighted the need for educators who work with Cambodian American parents to consider mothers' and fathers' beliefs systems and to identify personal and contextual resources to increase parents' involvement in children's education.

To sum up, the examined studies make reference to the following factors as determinants of father involvement: fathers' and mothers' education, families' income level, residencial status, race-ethnicity, characteristics of fathers and children, mother-father relation and social support for positive involvement. Indeed, although the studies in this field are characterized by a great heterogeneity, there is a general agreement in the literature about the necessity to consider how personal (e.g., mental health, child temperament, personality), interpersonal (e.g., marital quality, coparenting), and contextual factors (e.g., social support, culture) influence one another on their impact on father involvement.

### Programs to Increase Father Involvement

As seen, although many studies highlighted that mothers were engaged in their children's education more frequently than fathers (Duursma, 2014; Kim and Hill, 2015; Baker, 2018), at the same time, there is strong evidence that fathers can play a unique role in children's cognitive skills development (Flouri and Buchanan, 2004; McBride et al., 2005, 2009; Roopnarine et al., 2006; Saracho, 2007b; Downer et al., 2008; Fagan and Lee, 2012; McWayne et al., 2013; Duursma, 2014; Jeynes, 2015; Kim and Hill, 2015; Baker, 2017, 2018).

Consequently, in recent years, there was an increase in studies focused on the efficacy of father involvement interventions. Indeed, among the 40 selected articles, the focus most investigated, after the relationship between father involvement and children's outcomes, was the benefits and strategies that can improve father involvement in children's education.

For example, Lipscomb (2011) reviewed various intervention types and found that different programs exist, each designed for specific population groups (programs related to incarcerated fathers, minority ethnic groups, and fathers with low income or literacy skills). The following are some examples: The My Baby's Father (MBF) Involvement Model; Dads at School; the Alliance of Concerned Men (Abridging); 100 Black Men; Long Distance Dads; and the Incarcerated Fathers Program. The study of Saracho (2008) can be considered an example of research on the evaluation of an intervention specifically related to fathers with low literacy. The results demonstrated that, when fathers improve literacy strategies, this can help and support their children's literacy skills development.

There are other important contributions in the same direction. Baskwill (2008) identified three main benefits after participating in the Picture It, Dads! (PID) literacy initiative: (1) fathers improved their literacy skills; (2) participants increased their knowledge about the relevance of father involvement in children's well-being; and (3) men acquired a wide range of dad-friendly strategies and increased their ability to engage with their children in learning and intellectual activities.

Chawla-Duggan (2006) identified two ways in which father development workers (FDWs) can help improve men's involvement with their children's education: (a) by encouraging fathers within the group to increase their self-confidence and (b) by helping fathers use both indirect and direct learning approaches to improve their children's intellectual abilities. Potter et al. (2012) examined the findings of fathers' participation in the Father's Transition Project (FTP), which aims to increase the involvement of fathers who live in deprivation areas in children's education during their transition to school. The more successful strategies in engaging fathers were: the focus on strengths rather than weakness, cooperation within the group, constant follow-up, the use of male activities, and mothers' engagement. Moreover, the main program participation benefits can be identified as follows: closer relationships with their children and a higher level of father involvement in playing and in learning activities.

### Definition and Measurement of Father Involvement and Children's Cognitive Skills

#### Definition and Measurement of Children's Cognitive Skills

Most of the reviewed studies examined children's academic/student achievement, cognitive skills, and literacy skills. Regarding the studies that focused on cognitive skills, on one hand, some that assessed memory, vocabulary, problem-solving, enumeration, and the competence to form generalizations and classifications used the Bayley Short Form (BSF) or the Bayley Mental Development Index (MDI)^1^. On the other hand, other studies about cognitive skills investigated particular math and reading skills with the Woodcock-Johnson Psycho-Educational Battery (WJ): this measure is used in the studies that focused on academic achievement as well. Moreover, the studies that focused on father involvement and children's literacy skills assessed the relationship between the amount of time fathers spend reading books to their children and developing reading skills (Ortiz, 2000; Saracho, 2008). For instance, in his review, Saracho (2007a) found that fathers support and increase their children literacy skills by reading books, involving children in book discussions, recording which books have already been read, and stimulating children to enhance their reading (Saracho, 2007b).

#### Definition and Measurement of Father Involvement

To address the way father involvement is studied in the different researches, it is necessary to deepen the construct's characteristics: Table 4 shows that there are many types of father involvement and, for each, there is no single definition or component. Father involvement is a multidimensional construct that has been conceptualized and measured in various ways. For example, 40% of the articles focused on “father involvement” in general, defining and measuring the construct differently, without any other specification (e.g., related to school, home, etc.). Most of the studies used self-reports (40%), and only a few utilized interviews and observations (30%) or mothers' reports (10%); however, each self-report focused on different aspects of father involvement. For instance, Baker (2017) used a self-report questionnaire to investigate the frequency (1 = “never” to 5 = “always”) of fathers' participation in warm interactions and in three home learning activities with their children. Furthermore, the kind of control/discipline that fathers used with their children was assessed with a 5-point Likert scale, where 1 = “not at all like me” and 5 = “exactly like me.”

**Table 4:** Type of construct.

	**Construct**	**Frequency**
	***n*.**	**%**
Father involvement	15	47.5
Father involvement at school	4	10
Father involvement at home	1	2.5
Father involvement in education	4	10
Early father involvement	5	12.5
Engagement	11	27.5
	40	100

In another study, Tan and Goldberg (2009) investigated parental school involvement using a 26-item scale adapted from Tan and Goldberg (2009) About Parental Involvement.

It aimed to assess how frequently mothers and fathers are engaged in four different kinds of school/educational-related activities: direct school involvement, homework involvement, interpersonal involvement, and extracurricular activity involvement.

Each item was evaluated using a 5-point scale, where 1 = “never” and 5 = “always.”

These two studies are useful for understanding the great difference in the way in which the construct of father involvement is defined and measured.

## Discussion

### Growing Interest in the Role of Father Involvement in Children's Education

The first aim was related to the progression of the amount and nature of the researches on this theme over the years. The findings suggest that, although each research used a different operational definition of the father involvement construct, in recent years there has been a wide and constant interest increase about this issue. Moreover, most of the examined articles were empirical studies and, in particular, a sizable number had a quantitative design.

### Fathers' Living Conditions

Regarding our second aim, the analysis of fathers' research samples revealed that most included biological and residential fathers: this trend may reflect that this sample type is easier to recruit than one comprised of non-residential and non-biological fathers.

Regarding the SES and the ethnicity of the families, the data highlighted how, in comparison with past researches, in recent years the literature on father involvement is starting to consider cultural differences and different educational and economic levels.

Indeed, while the most of past researches collected samples of Caucasian and middle-class men, failing to grasp the families' diversity, more and more articles, recently, include minority populations or diversified samples both in terms of the cultural level and the SES of the family (Black et al., 1999; Ortiz, 2000; Saracho, 2007a, 2008; Cabrera et al., 2011; Sun et al., 2018).

### Main Research Focuses

The third objective was to analyze the main focuses of the examined literature. The findings revealed that the main focus is the impact of father involvement on children's cognitive skills. The studies highlighted a positive association between father involvement and children's cognitive skills^2^ in early and middle childhood (Flouri and Buchanan, 2004; McBride et al., 2005, 2009; Roopnarine et al., 2006; Saracho, 2007b; Downer et al., 2008; Fagan and Lee, 2012; McWayne et al., 2013; Duursma, 2014; Jeynes, 2015; Kim and Hill, 2015; Baker, 2017, 2018). Moreover, it is important to underline that several studies highlighted that this positive association between father involvement and children's cognitive skills remains significant across ethnicity and SES (Downer et al., 2008; Jeynes, 2015; Baker, 2017). Instead, regarding the family household, some authors argued that this association is lower for children living in two-parent families than for children living with a single mother (Fagan and Lee, 2012), whereas others supported the strength of this association, regardless of the family characteristics (Coley et al., 2011). In line with these findings, Volling and Belsky (1991), in their study of multiple determinants of father involvement in dual- and single-earner families, found that even the fathers' personality characteristics had a significant impact on their responsibility for child care in single-earner but not in dual-earner families; contextual factors (e.g., marital quality and work) were potential influents in both dual and single families.

The study of father involvement in different families' households allowed a wider understanding of the direct and indirect pathways of the fathers' influence on their children's development. Regarding the direct patterns of influence, many studies found that father–school involvement was positively and directly associated with children's reading, math, and approach to learning (Baker, 2018). Moreover, fathers' SES (including education level and income) is uniquely and directly associated with children's cognitive skills (Cabrera et al., 2007; Malin et al., 2012). Finally, depending on the level of involvement, fathers can positively affect children's development even when they do not live with them (Cabrera et al., 2000) while, according to the well-known contribution of Amato and Gilbreth (1999) about non-residential fathers, the complete absence of a father is associated with less success in school and impaired cognitive function. Moreover, with respect to indirect effects, fathers impact their children through their financial responsibilities by influencing the quality of children's home experiences (Cabrera et al., 2009; Kolak and Volling, 2013). Another indirect effect may be largely attributable to harmonious family contexts: there is empirical support for the hypothesis that a positive marital quality is associated with positive parent–child relationships and child adjustment (Gable et al., 1994), whereas marital conflict is associated with maladjustment (Emery, 1994; Cummings et al., 2004).

However, although these findings highlight the various pathways of fathers' involvement in influencing their children's development, they do not explain how fathers and mothers are similarly and differently involved in their children's development, and how this could similarly or differentially affect child outcomes. On one hand, some authors, in stressing the differences between parents, noted that fathers give a unique contribution to their children's development—one that is different from the mothers'. Most of the articles highlighted that, although mothers showed higher levels of involvement compared to fathers (Duursma, 2014; Kim and Hill, 2015; Baker, 2018), there is great evidence that fathers' involvement had a positive association with their children's academic skills, demonstrating a unique influence provided by the paternal contribution. In line with these results, several researches showed that fathers are more likely than mothers to engage their children, especially sons, in rough-and-tumble play (Hossain and Roopnarine, 1994; Panksepp et al., 2003; Paquette et al., 2003), to encourage them in risk taking (Hagan and Kuebli, 2007) and dealing with scary experiences (Sandseter and Kennair, 2011).

On the other hand, other researches have focused on the similarities between mothers and fathers and found that children may benefit from parental support regardless of which parent provides it, as long as it is frequent and of high quality (Ryan et al., 2006; Cabrera et al., 2007). To conclude, given the evidence of both the similarities and differences in father– and mother–child relationships, Cabrera et al. (2014) suggested considering fathers and mothers as a complements to each other, where each person's behavior can help strengthen or weaken the bond between them. Consequently, sometimes fathers will enact roles played by mothers, and vice versa, in response to environmental conditions that require adaptation (e.g., both parents working, single-parent fathers). Given these findings, a model that attempts to capture the complexities of father involvement must consider contextual and individual factors that may move fathers to being more similar to or different from mothers. For this reason, the examined articles that focused on the determinants of father involvement tried to identify which possible risk and protective factors are related to fathering.

Although these studies are characterized by a great heterogeneity, there is a general agreement in the literature about the necessity to consider how personal (e.g., mental health, child temperament, personality), interpersonal (e.g., marital quality, coparenting), and contextual factors (e.g., social support, culture) influence one another on their impact on father engagement. A current model that tried to compile these factors, overcoming linear and static approaches considering the transactional and reciprocal nature of the father–child relationship, is the *Ecology of Father–Child Relationships: An Expanded Model*, developed by Cabrera et al. (2014). It considers fathers as part of dynamic systems characterized by interconnected relationships between and among caregivers and children and explains how these relationships evolve and change through time and social and contextual factors. This model also considers the personal, interpersonal, and contextual variables in determining the level of father involvement (Volling and Belsky, 1991) and the transactional and reciprocal nature of the relationship between fathers and children (Sameroff, 2010).

As a consequence, if future studies enable the achievement of a shared knowledge and understanding of such factors, then researchers and professionals will be able to enhance father involvement through specific programs based on such protective and risk factors. Indeed, a sizable number of articles focused on the effectiveness of programs to improve paternal engagement. It is possible to discriminate two kinds of programs: one aimed toward samples with specific characteristics (e.g., Dads at School, 100 Black Men, Long Distance Dads, Incarcerated Fathers Program, programs for fathers with low-literacy skills); the other concerns programs that can be applied to a more generic sample (e.g., Father-to-Father Mentoring Program, Father in Training (FIT): Empowering Men to Become Better Fathers).

In the end, it is possible to summarize that, although the literature about father involvement is characterized by a great heterogeneity, there is a general agreement about the necessity to consider how personal (e.g., mental health, child temperament, father's personality, father and mother level of education,), interpersonal (e.g., marital quality, coparenting), and contextual factors (e.g., social support, culture) influence one another on their impact on father involvement.

### Definition and Measurement of Father Involvement and Children's Cognitive Skills

The last aim of the present review concerns the operational definition of the construct and the measurement instruments used to assess father involvement and their children's cognitive skills. Regarding the assessment of cognitive skills, the literature is quite heterogeneous: on one hand, some studies examined cognitive skills by evaluating problem-solving, memory, math ability, vocabulary, and competence to create generalization and categorization (Fagan and Lee, 2012); on the other hand, other studies investigated, in particular, math and reading skills as well as the studies focused on “academic/student achievement” (Baker, 2017, 2018). For this reason, it is clear that the most investigated children's outcomes are math and reading skills, also referred to as “cognitive skills” and “academic achievement.” As for father involvement, the selected researches used different types of constructs and tools to understand the father–child relationship, focusing on some aspects of father involvement and neglecting others.

Most of the selected researches used quantitative methods, in particular self-report questionnaires, usually developed or specifically adapted to assess mother–child relationships. In contrast, the use of interviews, observations, or diaries in qualitative studies to explore father–child relationships allow the assessment of more specific aspects. For instance, Keown and Palmer (2014), in their qualitative study, used semistructured interviews, which evaluated both the frequency and nature of parents' involvement with their children. Such heterogeneity related to the measuring instruments reduces the possibilities of comparing the results of each study: this remains one of the main limits in most empirical studies. Moreover, the use of only self-reported data does not make it possible to understand how the ecology of children's lives changes, how fathers interact with their children, how they engage in different activities, and what circumstances bring fathers into and remove them from their children's lives (Cabrera and Volling, 2019).

In conclusion, it could be argued that parental involvement assessment requires overcoming the traditional developmental models focused on dyadic interactions, usually mother–child, by using a broader multidimensional perspective, and thus a comprehensive methodological approach based on a developmental ecological system framework (Cabrera and Volling, 2019), to evaluate the father–child relationship in their family system, including a multidimensional, multi-informant assessment.

## Limitations

The main limitation across studies is related to generalizability. This aspect could reflect the recruitment process and the configuration of the samples: confined to residential, American and middle-class fathers. For example, in their study Fagan and Lee (2012) identified a limitation in the low response rate of non-resident fathers. Furthermore, given the increase of non-residential fathers in the general population, their involvement in study designs becomes more necessary.

The second limitation is related to measurement. Indeed, the ways in which father involvement is measured are criticized for more than one reason: (a) the simplicity of the construct's measurement (e.g., assessing the extent rather than the quality of interaction); (b) the wide variability of instruments; (c) instruments validation testing of the mothers; (d) the use of mothers' reports about fathers' involvement; and (e) the overuse of self-report questionnaires. In particular, the self-report measure of father involvement is not considered the best way to assess paternal engagement because it may reflect fathers' aspirations to look better than they might be (because of a social desirability bias), and they cannot grasp the dynamic and transactional nature of father–child interactions, which is a complex phenomenon that could be investigated across several dimensions (Cabrera et al., 2018; Cabrera and Volling, 2019). Thus, the integration of self-reported data with qualitative tools (e.g., observations) in father–child interactions should be preferred.

Further developments in evaluation procedures are needed for broader comprehension of fathers' involvement and their impact on their children's well-being: in fact this limitation on the source of information (e.g., parent report) could have and effect on the estimation of children's enjoyment of school and cognitive skills. The use of the same data source for both the independent and dependent variables may lead to overestimate the correlation between the variables, which, in turn, compromise the possibility of making causal inferences starting from parental involvement to understand the children's outcomes. For this reason, researches that utilize a multi-informant approach can be more informative compared to studies based on a single data source.

We must also take into consideration that, to avoid bias, we excluded the articles involving clinical samples, which would need to be addressed with a specific literature review.

A further limitation is about the design of the reviewed studies, since the majority of them used a cross-sectional design, which limits the inferences of causality.

Finally, in many studies, the reported interaction effects and sample sizes were quite small. However, although the great heterogeneity of the examined studies represented a limiting condition also for conducting the present review, it was possible to outline many important points in relation to the examined literature and to draw on future research directions.

## Implication for Future Researches

Given the previously discussed limits, the first implication for future researches is related to the need to expand the research samples, including more ethnic groups and different geographical contexts (e.g., suburban, urban, or rural areas)and low-income, non-resident, social, and step-fathers in the studies that are addressed to deepen the knowledge about father involvement. This is necessary because it could enhance both the internal and external validity of the existing studies about father involvement in the literature. Indeed, parenting occurs in a social context (Bronfenbrenner, 1979) and, for this reason, fathering patterns may vary by race or social context.

Another implication for future researches is related to the necessity of extending and deepening the conceptualizations of father involvement in children's cognitive skills development, not focusing exclusively on fathers engagement. Currently, there is vast evidence in the literature that father involvement is a multidimensional construct influenced by personality, family history, child characteristics, marital quality, and the father's sociocultural context (Volling and Belsky, 1991). For this reason, the use of one single source of information and one method of measurement of father involvement is reductive, and there is a necessity for studies to incorporate multiple informants and methods (e.g., observations, surveys carried out by both fathers and mothers, diaries) in the assessment of paternal involvement (Cabrera et al., 2018; Cabrera and Volling, 2019). Furthermore, future research should create and validate measures of parenting practices for both low-income and minority families and use longitudinal designs to better understand the association between father involvement and children's outcomes.

In conclusion, the consideration of the above indications in future research designs could make it possible to increase the knowledge about mothers' and fathers' involvement in their children's cognitive skills development.

## Author Contributions

LR, GG, and AC took overall responsibility for the creation of the frame used in this review and the selection of the papers. TT, LC, EG, and PB searched for the articles discussed in the review. LR and AC supervised the entire work. All authors were involved in the discussion, the write and the revision of the manuscript and they gave the final approval of the version to be published.

### Conflict of Interest

The authors declare that the research was conducted in the absence of any commercial or financial relationships that could be construed as a potential conflict of interest.

## Footnotes

^1^The MDI is the most used measure to assess toddlers' cognitive development, in which high scores reveal high levels of cognitive development.

^2^Further ahead, we will discuss the construct of the operational definition of “cognitive skills” which, in some articles, is also referred to as academic/student achievement.
